# Road Traffic Accidents, Climbing Frames, or Trampolines: What Harms Children in the 2020s?

**DOI:** 10.7759/cureus.35781

**Published:** 2023-03-05

**Authors:** Konrad Schuetze, Carlos Pankratz, Sabine Schütze, Jasmin Zieger, Florian Gebhard, Raffael Cintean

**Affiliations:** 1 Orthopedic Trauma Surgery, University Hospital of Ulm, Ulm, DEU; 2 Obstetrics and Gynaecology, University Hospital of Ulm, Ulm, DEU

**Keywords:** pediatric trauma protocols, pediatrics emergency, trauma critical care, pediatric injuries, trampoline, road traffic injuries, trauma pediatric, prevention, mechanism of injury, pediatric fractures

## Abstract

Background

In the modern Western world, activities and the daily routine of children have changed over time. Detailed analyses of the mechanisms of injuries and current fracture patterns in children are rare. The aim of the study was to elicit and investigate the most dangerous leisure and sporting activities leading to fractures in children today.

Materials and methods

This is a retrospective study focusing on children that were treated in a level-one trauma center in Germany between 2015 and 2020. All children who were 14 years of age or younger and suffered a traumatic injury treated in our emergency department were included in this study. From the database, mechanisms of injury, type of injury, age, and gender were analyzed.

Results

The study included 12508 patients, including 7302 males and 5206 females. Among the 10 most common mechanisms of injury were collisions (8.6%), falls (7.7%), injuries while playing (6.1%) or while running or walking (5.9%), soccer (5.9%), bicycle accidents (3.8%), and trampoline falls (3.4%). Road traffic accidents involving passengers or pedestrians caused 3.3% of the injuries but were the most common cause of death. The most common mechanisms of injury causing a fracture were falls, playing soccer, and bicycle accidents. Sorting the mechanisms of injury by the percentage that caused a fracture, the most dangerous activities were falling from heights above 2 meters, skiing and snowboarding, climbing and bouldering, skateboarding, and horseback riding. In the five-year study period, four out of six children died due to road traffic accidents.

Conclusion

Injured children must be provided with the best quality of care 24/7 in orthopedic trauma departments and have to be kept as a focus in the training of orthopedic trauma surgeons. Road traffic accidents are still the main cause of death in children, but they are overall less common. Falls and sports activities are the most likely to cause a fracture.

## Introduction

Unintentional injuries are pretty common in children and adolescents. In Germany, accidents are the most common cause of hospitalization for children older than five years and the second most common cause for children under five years [1]. Unintentional childhood injuries are a public health burden with a huge economic burden due to indirect costs like parental pay loss during injury-related increased childcare and direct costs like medical fees due to hospital treatment [2,3]. Kopjar et al. showed that children younger than 14 accounted for 31% of all unintentional injuries in Norway [2], and a recent study from China estimated the cost of fracture treatment for each child at about 1500 USD [3]. Injury patterns and mechanisms vary between different countries [2-5] and over time [4]. In high-income countries, injuries during leisure activities are considerably more common compared to low-income countries, where traffic accidents and falls are more frequent [3,5]. In Germany, where the study was carried out, the most popular type of sport is soccer, followed by gymnastics and tennis [5]. About 57% of the kids in Germany ride their bikes or walk to school, compared to only 23% that were driven to school by their parents [6]. In comparison, only 25% of the children in the US walk or bike to school [7], and the most common sporting activities are basketball, American football, and baseball [8]. Therefore, an influence of cultural, economic, and geographical circumstances on injury patterns can be expected. The injury mechanism has changed over the years [9]. Compared to 1998, Fitzgerald et al. showed that trampoline injuries nearly doubled in the United States of America in 2017 [4]. In countries like Germany, road traffic accidents involving injured children are constantly falling in numbers due to extensive prevention [10]. Because of the changed injury patterns and mechanisms, this study tried to evaluate the most common fractures in the 2020s and the most common mechanisms of injury to allow targeted prevention.

## Materials and methods

Institutional and prior ethical committee approval of the ethics committee of the University of Ulm for the use of data in this study was obtained under the number 37/21-FSt/Sta. The study follows the "Strengthening the Reporting of Observational Studies in Epidemiology" (STROBE) recommendations for reporting observational cohort studies [11].

All patients aged 0 to 14 who were treated in the emergency department of a level-one trauma center between January 2015 and December 2019 were retrospectively reviewed. All patients were treated by an orthopedic trauma resident in the emergency department. Operative treatment was performed or supervised by an attending orthopedic trauma surgeon. Injury mechanism, type of injury, and type of treatment as well as demographic factors were recorded for all included patients. Age was classified into five groups. The age groups were: babies up to age one; toddlers between ages one and three; preschoolers between ages four and six; young schoolchildren from ages seven to 10; and young adolescents between ages 11 and 14. The type of injury was mainly divided into the categories of bruises, lacerations, fractures, distortions, joint dislocations, and burns. The study focused mainly on fractures, which were further divided by their localization. Only mechanisms of injuries with three or more cases were recorded. Mechanisms with fewer than two cases were combined as "miscellaneous". Also, in cases where the records were incomplete or unreadable, they were included in miscellaneous. Data analysis was performed with IBM Statistical Package for Social Sciences (SPSS) (V21.0) and Microsoft Excel (V16.3); demographic characteristics are described as numbers and percentages.

## Results

There were 7302 boys and 5206 girls included in the study. Divided according to their age, there were 27.9% young adolescents, 27.1% young school children, 19.3% preschoolers, 16.8% toddlers, and 8.8% babies. Children suffered most likely from bruises (41.2%), lacerations (23.4%), and fractures (21.6%). Distortions (10.3%), joint dislocations (2.6%), and burns (0.6%) were less likely. In 0.4%, there was no injury recorded or the injury was classified as other. Out of the 2703 children, 700 were treated operatively and 2003 conservatively. Excluded were 70 cases without documentation and 1182 cases that were categorized as "miscellaneous."

Mechanisms of injury

**Figure 1:**
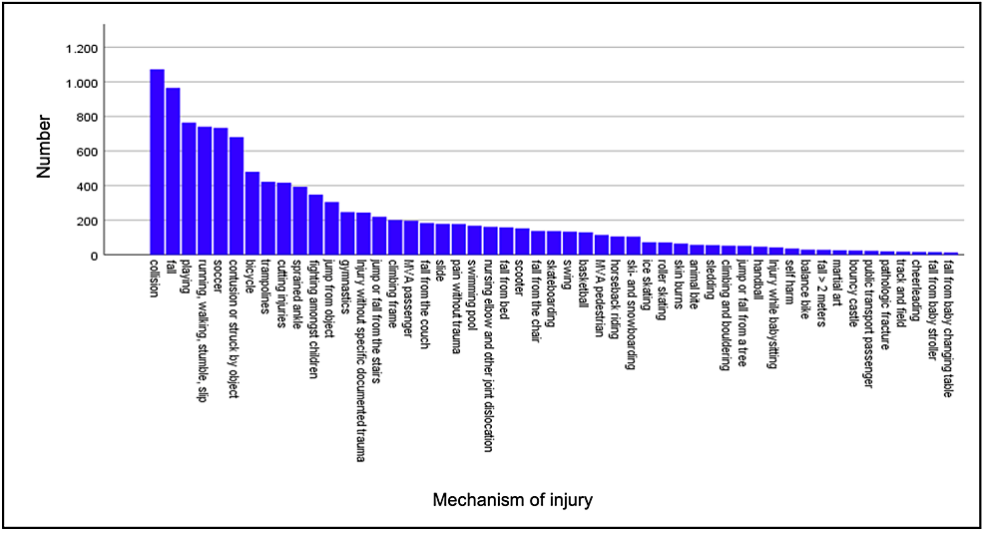
Injury mechanisms are sorted by number. MVA: motor vehicle accident

The most common mechanism of injury was collision with another child or object (8.6%), fall (7.7%), injury while playing (6.1%), or while running or walking (5.9%). After these common injury patterns, there are three very specific mechanisms in the top 10: injury while playing soccer (5.9%), bicycle accidents (3.8%), and trampoline falls (3.4). Suspected injuries like motor vehicle accidents as passengers (1.6%) or pedestrians (0.9%) meanwhile are in 17th and 29th place. In four cases, suspicion of child abuse was documented. All mechanisms of injury are shown in Figure 1.

Types of fracture

**Figure 2:**
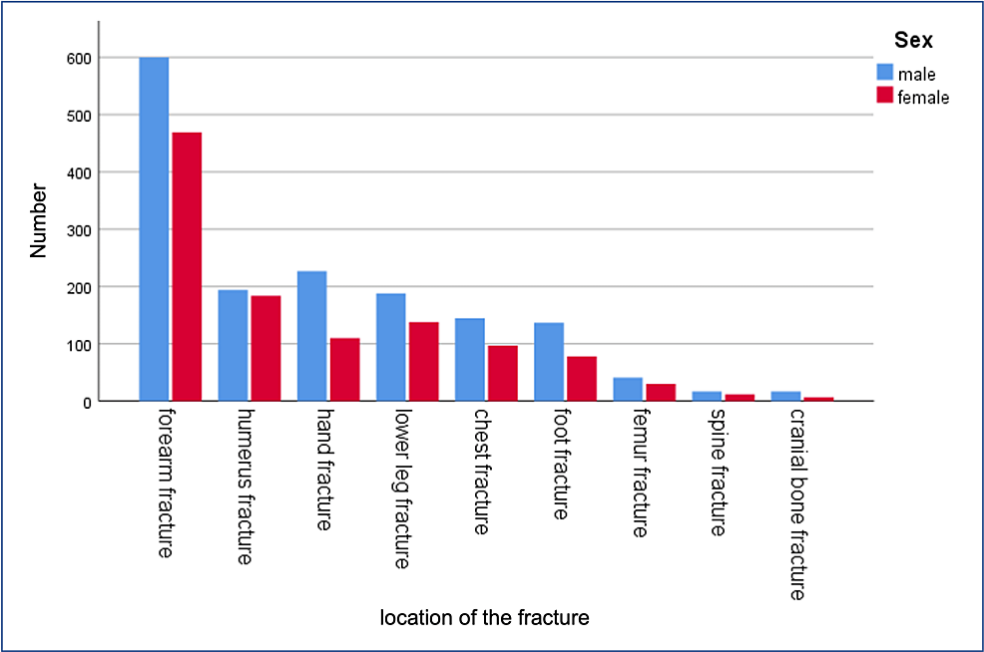
Fracture locations for boys and girls, sorted by number.

**Figure 3:**
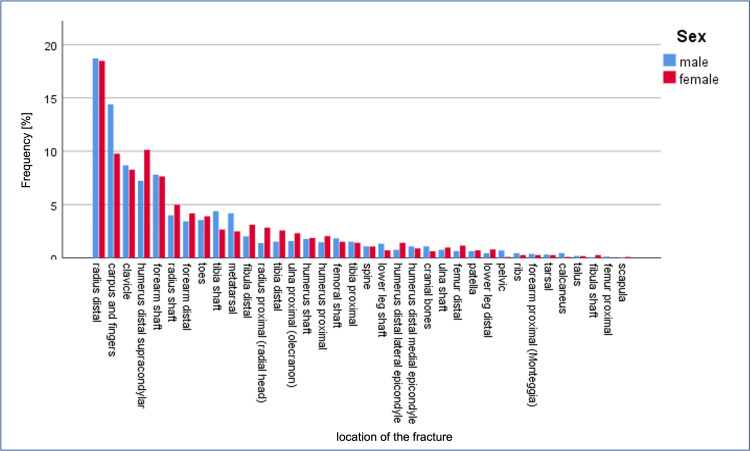
Detailed fracture locations for boys and girls, sorted by frequency.

Of the 2703 fractures, 51.2% were forearm fractures, 21.0% were humerus fractures, 12.4% were lower leg fractures, 6.0% were hand fractures, and 5.9% were femur fractures. Rare fracture localizations were foot (1.7%), chest (1.2%), spine (0.3%), and cranial bone fractures (0.3%). The most common of the 2703 fractures were distal radius fractures (18.6%), followed by carpus and finger fractures (12.5%), fractures of the clavicle (8.5%), supracondylar fractures (8.4%), and forearm shaft fractures (7.7%). While the spine was the fracture location in 1.1% of cases, the pelvis, ribs, and scapula were less common, at under 0.5%. Fracture categories sorted by numbers are shown in Figure 2. Detailed fracture locations sorted by frequency are shown in Figure 3.

Mechanisms of injury that resulted in a fracture

**Figure 4:**
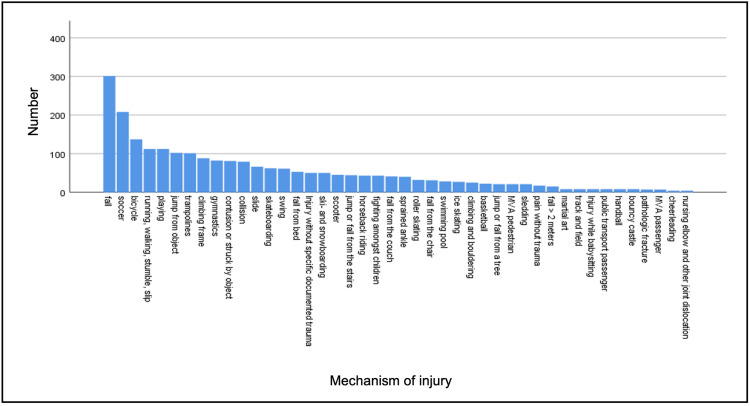
Mechanisms of injury resulting in a fracture, sorted by number. MVA: motor vehicle accident

The most common mechanism of injury resulting in a fracture was a fall (12.8%), followed by an injury while playing soccer (8.8%), and bicycle accidents (5.8%). All mechanisms of injury resulting in a fracture, sorted by their number, are shown in Figure 4.

**Figure 5:**
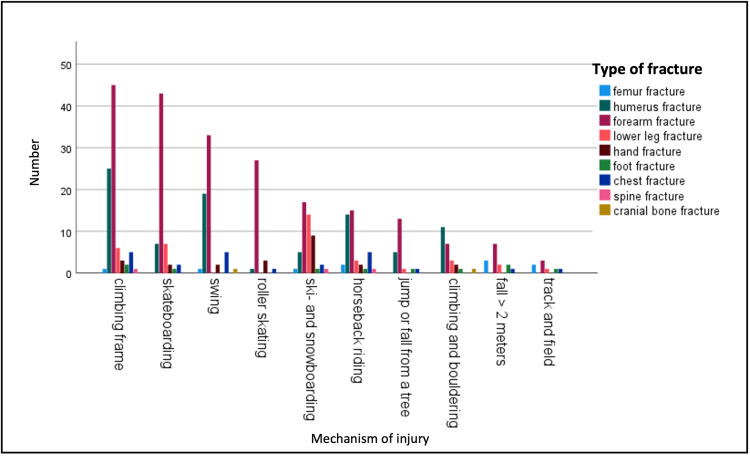
Top 10 mechanisms of injury most likely to result in a fracture, with their fracture location.

The mechanism of injury with the highest probability of suffering a fracture was a fall from heights above 2 m, with a diagnosed fracture in 53.6% of these cases. This was followed by skiing and snowboarding (47.6% with a diagnosed fracture), climbing (47.2% with a diagnosed fracture), falling from the swing (45.5% with a diagnosed fracture), and skateboarding (45.3% with a diagnosed fracture). The most dangerous activities and the resulting fractures are shown in Figure 5.

## Discussion

This study focused on current fracture patterns and mechanisms of injury resulting in fractures in pediatric patients. The data are from a German city with a population of over 100,000, where only one level-one trauma center treats pediatric injuries. Thus, the study provides solid data for current injury mechanisms and fracture patterns in children.

The age group most at risk for injury was young adolescents between the ages of 11 and 14, with more injuries than even toddlers and babies combined. This is in line with the findings of Bradshaw et al., who compared low- and high-income countries in their study [12]. Through all age groups, boys were more often injured than girls. This is in line with several studies from Iran, Spain, the United Kingdom, and the United States of America [12-15]. Reasons often cited in the literature include greater exposure to risky sports activities as well as different gender-related behavior patterns [16,17]. The most common fracture locations were the distal radius, carpal and finger joints, clavicle, supracondylar humerus, and forearm shaft, which is therefore comparable with other epidemiological studies [18]. A gender comparison showed more carpal and finger fractures in boys and more supracondylar fractures in girls.

Collisions and falls were the most common mechanisms of injury, and falls were also the most common cause of fractures in our study. Multiple studies evaluated falls as one of the most common causes of injury in children [12-14,19,20]. Most studies only distinguished between low and high falls. Only Beck et al. distinguished further and evaluated a comparable rate of 3.2% for falls from furniture and beds [20]. Interestingly, we were able to identify a fall from the changing table as the cause of injury in only 0.1% of all patients examined, and no serious injury such as a fracture was detected. This is in line with all of the mentioned studies.

A general presumption is that most accidents involving children happen on the road. In this study, the incidence of road traffic accidents involving passengers and pedestrians was low and evaluated in only 3% of the cases. Accidents involving pedestrians occurred in 1.0% of all cases and resulted in a fracture in 16.6% of these cases. Especially in low-income countries, road traffic accidents are a major trauma mechanism harming children [21-23], but they also accounted for 23.7% of injuries in Spain [14] and 60% in the USA [24]. Compared to these studies, we included every child that presented in the emergency room, while most other studies only included severely injured patients with an injury severity score greater than 16 [19,24]. Furthermore, by excluding patients over 14 years, we had no motorcycle accidents with adolescent drivers. Still, in the study period, six severely injured patients with an injury severity score greater than 16 were treated. Four of them were road traffic accidents as pedestrians (three were hit by cars; one was hit by a fallen traffic light); one fell while horseback riding; and one was buried under a stone slab. All deceased patients were injured in a traffic accident and died due to severe traumatic brain injuries. While road traffic accidents are less common compared to sports-related injuries or falls, they are still the main cause of death among children. Furthermore, only accidents that happened on a road were counted as road traffic accidents in our study. Accidents with bicycles, pedal scooters, or walking bikes made up 5.9% of injuries and were not counted as road traffic accidents. All three accounted for 8.2% of the fractures, with the bicycle being the overall third most common cause of a fracture in children. About 25% of the children in Germany ride their bikes to school, which might explain this high rate. In 1995, sports-related injuries were more common and evaluated as mechanisms of injury in children (15.9%). This is substantially higher than in the studies of Navascues (4.2%) and Snyder et al. (8.1%) [14,25]. In line with the study of Randsborg et al., most injuries happened while playing soccer (6.5%), which was also the second most common mechanism resulting in a fracture [26]. Jumping on the trampoline (3.7%), gymnastics (1.9%), or skateboarding (1.2%) were pretty common too. In line with the study of Berger et al., we showed how trampoline accidents are becoming more common and often result in a fracture [27,28]. If we look more closely at the percentages of children with fractures in the present study, we find that the most dangerous sports, with over 40% of the cases presenting with a fracture, were climbing and bouldering (47.2% of the cases suffered a fracture), skiing and snowboarding (47.6%), track and field (44.2%), skateboarding (45.3%), and horse riding (41.0%). The targeted prevention of road traffic accidents had a significant effect [9,10], but as underlined by our results, physical education is also very important in the development of children. In dangerous activities like horseback riding or skiing, protective gear should be worn at all times.

The study has certain limitations. Due to the handwritten documentation, some mechanisms of injuries could not be evaluated, and due to the manifold of mechanisms, we excluded cases with fewer than three occurrences. Furthermore, the very frequent mechanisms of collision and falls were often not described in detail and could therefore not be categorized further. The study was carried out in a major German city and only included children who were treated in the hospital. So more severe injuries might be overrepresented because smaller cuts and bruises are often treated by a family physician. Also, traditional German sporting and leisure activities like soccer, horseback riding, and trampoline jumping might be overrepresented compared to other countries. As a strength, this study gives a detailed assessment of today's fracture patterns and mechanisms of injuries in a developed Western country.

Overall, the conservative and operative treatment of injured children must always remain the focus of the orthopedic trauma surgeon, as the trauma mechanism might change but the fracture incidence and severity stay the same. In a German city of over 10,00,000 inhabitants, we treated roughly 2500 children per year in the emergency department. About 540 fractures a year were diagnosed, and 140 children a year had to be treated operatively.

## Conclusions

The treatment of pediatric injuries continues to be a challenge for the treating physician and should be performed by a specialized trauma center, especially for severe injuries. While the causes of injuries may have changed, single bone fractures in particular continue to be a common injury in children. Leisure activities such as rock climbing, skateboarding, track and field, and horseback riding figure prominently in the cause. Traffic accidents with serious injuries, although rare, are the leading cause of fatal outcomes.
